# Molecular Profiling of Glioblastoma Patient-Derived Single Cells Using Combined MALDI-MSI and MALDI-IHC

**DOI:** 10.1021/acs.analchem.4c03821

**Published:** 2025-02-11

**Authors:** Kasper K. Krestensen, Tim F. E. Hendriks, Andrej Grgic, Marleen Derweduwe, Frederik De Smet, Ron M. A. Heeren, Eva Cuypers

**Affiliations:** † The Maastricht MultiModal Molecular Imaging (M4I) institute, Division of Imaging Mass Spectrometry (IMS), Maastricht University, 6229 ER Maastricht, The Netherlands; ‡ Laboratory for Precision Cancer Medicine, Translational Cell and Tissue Unit, KU Leuven, 3001 Leuven, Belgium

## Abstract

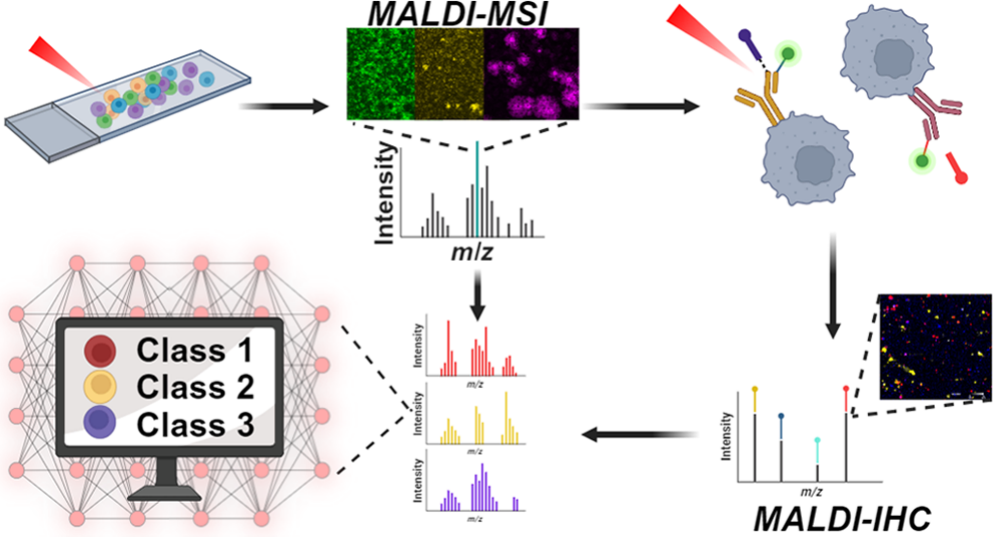

In recent years, mass spectrometry-based imaging techniques have improved at unprecedented speeds, particularly in spatial resolution, and matrix-assisted laser desorption/ionization (MALDI) mass spectrometry imaging (MSI) experiments can now routinely image molecular profiles of single cells in an untargeted fashion. With the introduction of MALDI-immunohistochemistry (IHC), multiplexed visualization of targeted proteins in their native tissue location has become accessible and joins the suite of multimodal imaging techniques that help unravel molecular complexities. However, MALDI-IHC has not been validated for use with cell cultures at single-cell level. Here, we introduce a workflow for combining MALDI-MSI and MALDI-IHC on single, isolated cells. Patient-derived cells from glioblastoma tumor samples were imaged, first with high-resolution MSI to obtain a lipid profile, followed by MALDI-IHC highlighting cell-specific protein markers. The multimodal imaging revealed cell type specific lipid profiles when comparing glioblastoma cells and neuronal cells. Furthermore, the initial MSI measurement and its sample preparation showed no significant differences in the subsequent MALDI-IHC ion intensities. Finally, an automated recognition model was created based on the MALDI-MSI data and was able to accurately classify cells into their respective cell type in agreement with the MALDI-IHC markers, with triglycerides, phosphatidylcholines, and sphingomyelins being the most important classifiers. These results show how MALDI-IHC can provide additional valuable molecular information on single-cell measurements, even after an initial MSI measurement without reduced efficacy. Investigation of heterogeneous single-cell samples has the potential of giving a unique insight into the dynamics of how cell-to-cell interaction drives intratumor heterogeneity, thus highlighting the perspective of this work.

## Introduction

Immunohistochemistry (IHC) has, since its inception, remained the gold standard in the study of cellular structure in histological tissue samples. Using antibodies labeled with fluorescence tags it is possible to identify single cell types in a tissue and visualize specific biomolecules in their native cellular location.^1,2^ Current microscopy instruments routinely allow for subcellular resolution, enabling precise pathological annotations and IHC is therefore an invaluable tool in clinical biomedicine. Both the research- and diagnostic value of IHC is greatly increased by the fact that fluorescence microscopy allows for visualization of multiple targets in the same experiment.^3,4^ While multiplexing using fluorescence microscopy is possible, the upper limit of targets is already reached around eight targets, due to spectral overlap and cross-reactivity. Additionally, the overlap in excitation and emission bands between fluorophores, greatly reduces the specificity of the method, counteracting the positive aspects gained from using multiple fluorophores.^5^ Other multiplexing methods, such as PerkinElmer’s OPAL multispectral platform, t-CyCIF,^6^ and CODEX,^7^ rely on iterative workflows that involve the repeated addition and removal of numerous probes. These processes are therefore highly time-consuming and carry the risk of confounding results due to incomplete or unsuccessful cycles.^8^

As an alternative, matrix-assisted laser desorption/ionization (MALDI) mass spectrometry imaging (MSI) is an analytical tool enabling untargeted detection and visualization of biomolecules in their native tissue environment. By altering the matrix used for analyte extraction, MALDI-MSI can be used to detect an array of molecules including lipids, proteins and metabolites and thereby provide multiomics insights into the sample of interest.^9^ Recently, targeted analysis using the strengths of MALDI-MSI has gained a lot of focus with the implementation of MALDI-IHC.^10^ MALDI-IHC combines traditional immunohistochemistry and MALDI-MSI with the use of Miralys probes, where antibodies are linked to an ionizable photocleavable mass-tag (PC-MT). After the antibody binds to its epitope, the PC-MT can be released via ultraviolet (UV) illumination and measured with MALDI-MSI. Each antibody is conjugated to a PC-MT with a different molecular mass, enabling high multiplexing of proteins or glycans without the drawbacks of other multiplexing methods, by relying on MS detection. Currently, the highest achieved published plexity of MALDI-IHC is 27 different targets.^11^ Additionally, Miralys probes also have a fluorophore attached to the antibody, enabling further imaging with fluorescence of the stained samples.

In recent years, the achievable spatial resolution of MALDI-MSI, here defined as pixel size, has increased rapidly and measuring pixels down to 5 × 5 μm is now routine. The increased spatial resolution enables analysis of even more biological sample types, as subcellular pixel sizes allow measuring of single cells,^12,13^ and in some cases even cellular compartments.^14^ The ability to measure single cells opens opportunities toward obtaining cell-specific molecular profiles, using MALDI-MSI, depending on specific lipid, peptide or metabolite composition detected.^15,16^ Increasing amounts of research is being done on dispersed single cell populations which allows for a clear differentiation between single cells. The use of mixed cell type cultures has the ability to mimic simple cell–cell interactions and thereby provide information on the complex dynamics observed in tissues.^17^ Furthermore, it was shown that profiles from two-dimensional (2D) cell cultures can be used to build models that are able to detect cell types back in a tissue environment and thereby aid in personalized therapy testing if patient-derived cell lines are used.^15^ Untargeted imaging of larger molecules has also become possible, with recent developments in nanospray desorption electrospray ionization enabling detection of different proteoforms on single-cell level.^18,19^ Spatial omics is the multimodal approach of obtaining spatial tissue information and molecular characteristics at the same time.^20,21^ Within the field of MSI, this is often achieved by detection of multiple molecular species with MSI or combining the untargeted and targeted approaches through for example MALDI-IHC or even targeted liquid chromatography–mass spectrometry (LC-MS).^22^ This combined approach can help enhance our fundamental biological understanding of tissues by simultaneously detecting both predefined analytes and novel molecular species in their native microenvironment and at high spatial resolution.

However, a complication that follows with an increase in spatial resolution, is a quadratic reduction in sensitivity due to the pixelated nature of sample ionization in MALDI-MSI. As the pixel size decreases, less material is ablated, resulting in a lower number of total ions generated. Consequently, a tendency to mainly see high-abundant species or species that are more easily ionizable is observed.^14^ To mitigate these challenges, sample preparation is of utmost importance to ensure proper interaction between the matrix and the analyte, as well as ensuring that results are stable and reproducible.

**Figure 1:**
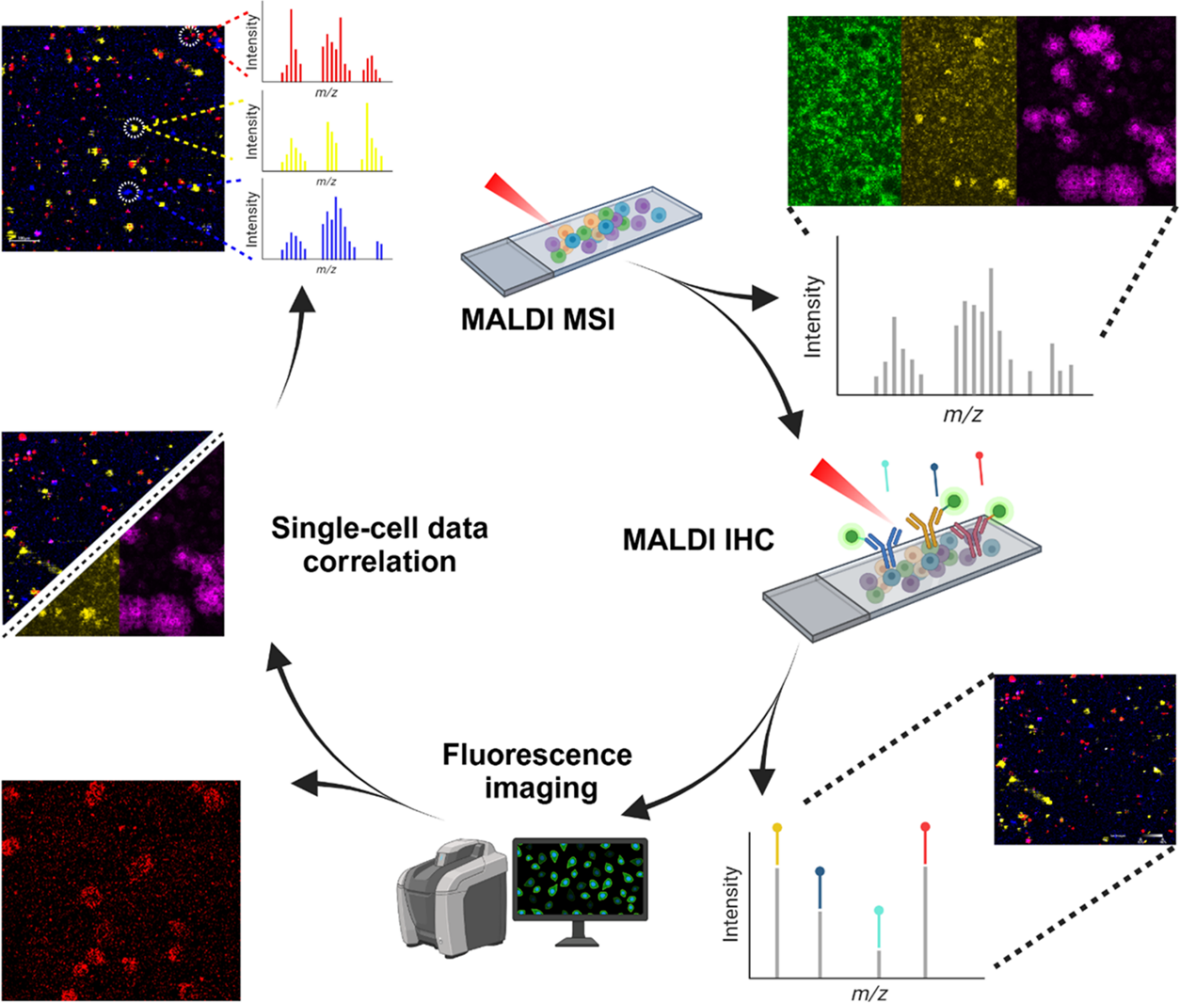
Optimized workflow for single cell MALDI-IHC and molecular profiling on a single slide. First, lipid profiles are obtained from the PDCL GBM single cells using high-resolution MALDI-MSI. After matrix removal, the cells are stained with cell-specific MALDI-IHC probes, for cell characterization. Fluorescent markers on the probes allow for optical confirmation of cellular locations. Finally, MALDI-IHC and MALDI-MSI images can be overlaid to coregister single cells between measurements and extract cell type specific lipid spectra. Created in BioRender.com.

Here we propose a workflow for measuring single patient-derived cells (PDCL) from glioblastoma (GBM) tumor samples (Figure 1). These cell cultures are grown without induction of specific cell types resulting in a heterogeneous mix of cells ranging from GBM cells, originating from glial cells like astrocytes, to healthy glial cells and neurons.^23^ The cells were imaged, first with high-resolution MALDI-MSI to obtain a lipid profile, followed by staining and imaging with MALDI-IHC to visualize cell type specific protein markers. The two modalities were then overlaid to provide cell type specific lipid profiles. Furthermore, we investigated the effect of the initial MSI measurement and the required sample preparation on the subsequent MALDI-IHC measurement.

## Materials and Methods

### Chemicals

Water (HPLC and ULC/MS grade), ethanol, acetone and chloroform were obtained from Biosolve BV (Valkenswaard, The Netherlands). α-Cyano-4-hydroxycinnamic acid (CHCA), phosphate-buffered saline, acetic acid, citric acid, bovine serum albumin, ammonium bicarbonate, 2,5-dihydroxybenzoic acid (DHB) 98% and ammonium phosphate monobasic were obtained from Sigma-Aldrich (St. Louis, MI). Tris-buffered saline, sodium citrate and octyl β-d-glucopyranoside were obtained from Merck KGaA (Darmstadt, Germany). Mouse and rabbit serum was obtained from Jackson immunoresearch (Ely, U.K.). Miralys probes were obtained from Ambergen (Billerica, MA).

### Samples

Fresh tumor tissue was collected from patients undergoing surgical resection at UZ Gasthuisberg, with all patients providing informed consent (S59804). Upon receiving the tissue samples at the LPCM lab, they were immediately processed for establishment of patient-derived GBM stem cell culture (S61081), as previously described.^23,24^

For the results shown here, cells from one donor (female, age = 45) are highlighted. Approximately 10^6^ cells (∼1.5 × 10^5^ cells/mL) were grown on slides suitable for MALDI-MSI measurement, indium tin oxide (ITO, CG-40IN-S115, Delta Technologies) glass slides coated with poly-l-lysine, as previously described.^15^*N* = 4 cell covered ITO slides were prepared and measured with MSI and MALDI-IHC. Cells were frozen in liquid nitrogen and stored at −80 °C until measurement.

### Mass Spectrometry Imaging

Prior to MALDI-MSI, the cells were removed from storage and kept in a desiccator box at room temperature for 30 min to avoid molecular delocalization during thawing. Fiducial markers were applied around the areas to be measured for coregistration. As matrix, 50 mg DHB in 1.5 mL acetone was sublimated onto the slide using an HTX Sublimator (HTX technologies, Chapel Hill) at 160 °C for 200 s. The sublimation tray was preheated to 60 °C. For evaluation of the effect of MALDI sample preparation and MALDI-MSI, respectively, on the subsequent MALDI-IHC measurement, one-third of the slide was covered during matrix application, to have a region which had undergone no prior intervention.

The cells were imaged on a rapifleX MALDI Tissuetyper instrument (Bruker Daltonik GmbH, Bremen, Germany) with a pixel size of 10 × 10 μm^2^, in positive ion-mode and a mass range from *m*/*z* 600–1340 for lipid detection. Red phosphorus was spotted on the slide for external calibration.

### MALDI-IHC

The methods were based on previous work and adapted for single cell applications.^10^ Briefly, the cell slides were prepared for staining by first removing any remaining matrix by washing in −80 °C acetone for 3 min, 2 times. All washing steps were conducted in separate glass Coplin jars. The slides were then dried for 10 min in a desiccator and fixated in 1% PFA for 30 min, followed by a PBS wash for 10 min, an acetone wash for 3 min, 2 times, and a wash in Carnoy’s solution for 3 min. Slides were then rehydrated with an ethanol series of 100% ethanol for 2 min, 2 times, 95% for 3 min, 70% for 3 min and 50% for 3 min. Finally, slides were washed with TBS for 10 min. Next, the cells were prepared for staining by antigen retrieval in citrate buffer at pH = 6, using a Retriever 2100 (Aptum Biologics Ltd., Rownhams, U.K.) for 20 min at 121 °C. The retriever body with slides was removed and cooled in an ice bath for 5 min, after which half of the retrieval buffer was replaced with HPLC grade water and the body was placed back in the ice bath for 5 min. This was repeated 2 more times and slides were then washed with TBS for 10 min. To limit the use of blocking buffer and antibody solution, the region to be stained and measured was surrounded using a hydrophobic PAP pen (Sigma-Aldrich, St. Louis, MI). Each region was then incubated with 150 μL blocking buffer for 1 h. Excess blocking buffer was carefully removed from the slide and cells were then incubated overnight (18–21 h) with 150 μL antibody solution at 4 °C in a humidified dark chamber, to prevent evaporation of solution and bleaching of fluorophores. An overview of the used antibodies can be seen in Supporting Table S1. From this point, slides were kept covered/in the dark at all times. After staining, the slides were washed in TBS for 5 min, 3 times, ABC for 10 s, and ABC for 2 min, 3 times, all while slightly agitating before drying them completely in a desiccator.

The peptide mass tags were cleaved off by UV illumination at 365 nm with a Phrozen UV curing lamp for 10 min (3 mW/cm^2^) prior to MS imaging. As matrix, 40 mg CHCA in 1.5 mL acetone was sublimated onto the slide using an HTX Sublimator at 180 °C for 360 s. The tray was preheated to 70 °C. Following sublimation, the slide was briefly dipped into an ammonium phosphate monobasic solution (0.5 mM) and dried vertically in a desiccator until fully dry.^25^ The stained cell regions were then imaged on a rapifleX MALDI Tissuetyper in positive-ion mode, with a pixel size of 5 × 5 μm^2^ and a mass range of *m*/*z* 820–1840. Red phosphorus was spotted on the slide for external calibration.

### Fluorescence Imaging

Fluorescence imaging by stimulated emission depletion (STED) microscopy was employed to confirm binding of the Miralys antibody probes to the cells. STED images were obtained using a commercial STED microscope (TCS SP8 STED, Leica Microsystems, Germany), equipped with a UV- and white-light laser. A Fluotar VISIR 25*X*/0.95 numeric aperture water immersion objective (Leica Microsystems, Germany) was used for imaging. Images were taken using a 592 nm excitation wavelength, a scan speed of 400 Hz with a 610–675 nm emission detection range respectively using gated hybrid detectors. The pixel size was approximately 0.91 μm (1024 × 1024 pixels), and 2-line averaging was performed.

Further, automated staining and imaging was done on the COMET platform. PDCL GBM single cell samples were stained with a GFAP marker. The stainings were performed as reported by Lunaphore in the literature.^26^

### Data Analysis

MALDI-MSI and MALDI-IHC images were visualized and analyzed using SCiLS lab 2024b (SCiLS GmbH, Bremen, Germany). MALDI-MSI images were RMS normalized and MALDI-IHC were TIC normalized. Average spectra from MALDI-MSI images were exported from SCiLS lab and imported into mMass software where peak picking was performed with the following settings: S/N threshold = 3, relative intensity threshold = 0.5%, picking height = 75, with baseline correction and deisotoping functions enabled. One-way ANOVA and *t* test calculations were done in R (Version 4.1). Cells were selected for analysis by creating ROIs around signals which were clearly separated out from other signals indicating that it is a lone standing cell. The cells selected contained between 5 and 15 pixels to avoid selecting too small areas but also too big areas which could represent more than one cell. The cutoff at 10 cells was selected based on the number of cells present per probe. For some of the low abundant probes (pTau, CD163, PVALB) it was difficult to select more than 10 cells. Lipid masses were matched and identified based on previous results with LC-MS/MS from GBM tissue, as described in.^27^

The classification model was created using the “training” and “classification” options in SCiLS lab 2024b. Between five and 12 cellular ROIs, corresponding with a specific class based on MALDI-IHC, were selected per class with repeated random subsampling at 15% used as cross validation parameter. Two separate MALDI-IHC measurements were needed to cover the entire MALDI-MSI measurement region, due to the difference in pixel-size. The classification model was trained on one of these MALDI-IHC measurement regions, while the classification itself was carried out on the other region.

## Results and Discussion

### Single Cell MALDI-IHC

#### Method Optimization

A number of adaptations were made to the recommended staining protocol to optimize the MALDI-IHC method for high-resolution, single cell measurements. First, to reduce potential delocalization or diffusion of molecules, all washes were performed at lower temperatures in ice-cold solutions. For measurements with pixel sizes down to 5 × 5 μm^2^ of single cells, any form of delocalization can be detrimental to the experiments and should be minimized, especially if the images are to be correlated across multiple modalities. Furthermore, when working with fresh frozen tissue, efforts to minimize delocalization are crucial as proteins in the tissue are left in their native state when unfrozen, compared to formalin-fixed paraffin-embedded tissue, where proteins are cross-linked in place by the formalin fixation. Previous work has shown that performing washing steps at freezing temperatures would reduce delocalization as compared to room-temperature washes.^28^

Second, the matrix application method was changed to achieve sufficient signal at 5 × 5 μm^2^ spatial resolution. Initial experiments were carried out with the recommended matrix application methods of either 2,5-DHB sublimation or automated CHCA spraying, both followed by recrystallization for increased analyte integration with the matrix and reduced crystal size.^22,28^ These experiments resulted in insufficient intensity, as only 2 out of 14 PC-MTs were detected at 5 × 5 μm^2^ pixel size (data not shown). To remove most of the matrix signal, which is usually obtained when sublimating CHCA, the slides were briefly dipped in ammonium phosphate monobasic after successful sublimation. This step significantly reduced signal from matrix clusters and resulted in high intensities for peptides even at 5 × 5 μm^2^ pixel size.^25^ Using this alternative matrix application method to visualize the PC-MT peptides improved the signal intensity enough to detect almost all stained markers and single cells from 10 out of 14 markers.

#### Effect of Pretreatment on MALDI-IHC Staining

**Figure 2:**
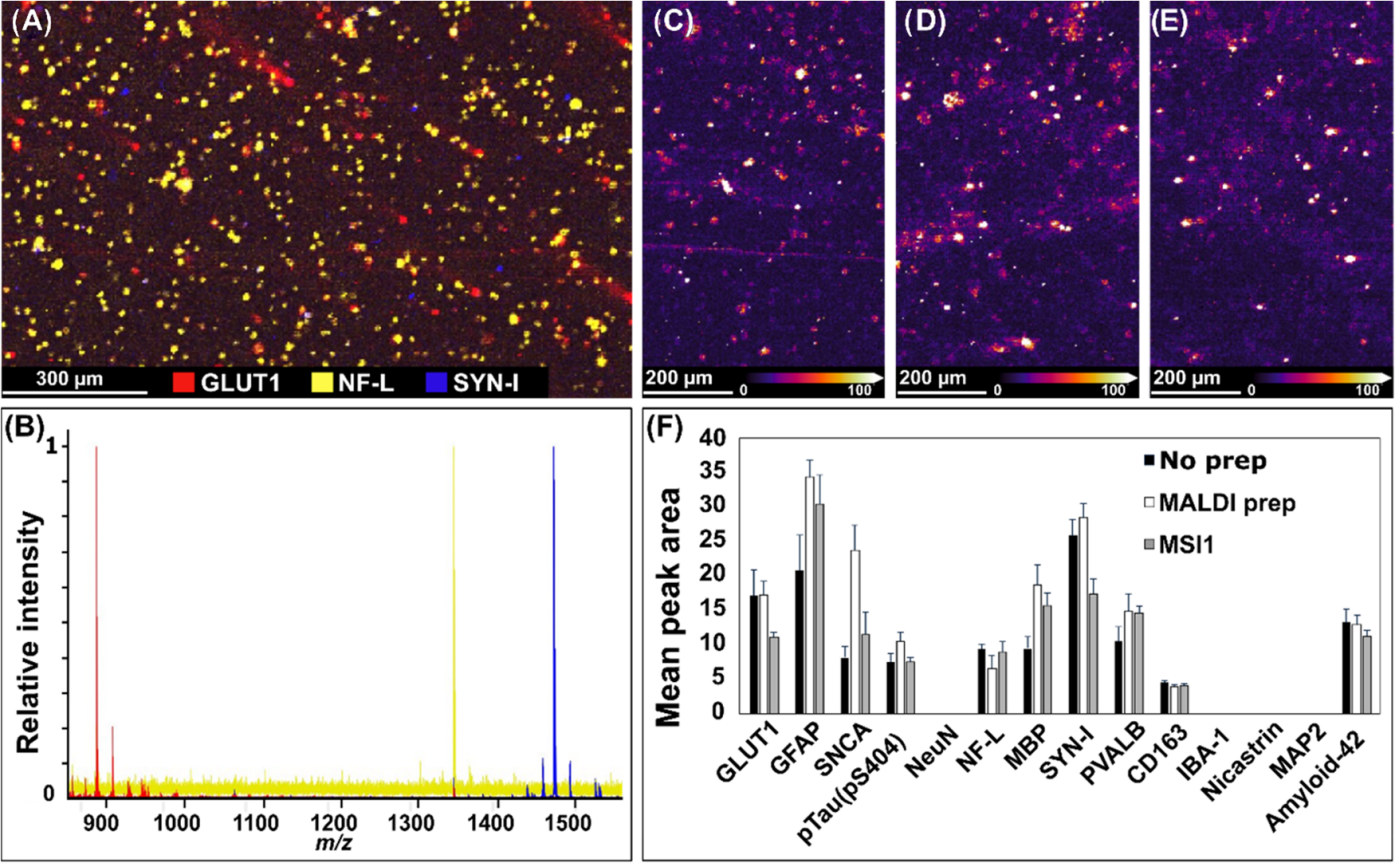
Patient-derived glioblastoma tumor cells stained with a 14-plex MALDI-IHC antibody panel. (A) Three different cell markers are highlighted: GLUT1, for GBM cells, in red, NF-L for neurofilaments in neurons, in yellow and SYN-I, as a synaptic marker, in blue. Pixel size = 5 × 5 μm^2^. (B) Single pixel spectra from three different cells stained by the markers shown in (A). Color-coded accordingly. (C–F) Shows the effect of prior measurements on the efficacy of subsequent MALDI-IHC measurements. (C–E) MALDI-IHC images of the mass corresponding with GLUT1 in cells that had undergone no prior treatment (C), cells that had undergone MALDI-MSI sample prep (D) and cells that had been measured with MALDI-MSI prior to the MALDI-IHC procedure (E). Pixel size = 5 × 5 μm^2^. (F) Mean peak areas, based on 10 single cells each, for each marker and condition. Each ROI, indicative of one cell, was manually determined. One-way ANOVA revealed no significant difference between the 3 groups (*p* = 0.73). Error bars indicate SE. Intensity of the markers NeuN, IBA-1, nicastrin and MAP2 were too low to detect single cells. All data presented in Figure 2 is obtained from *n* = 1 cell covered ITO slide as described in Supporting Figure S1. Replicate MALDI-IHC measurements can be seen in Supporting Figure S2. Data to support Figure 2F can be seen in Supporting Tables S2 and S3.

The same, identical cells needed to be measured twice, first with MALDI-MSI (lipids) and then with MALDI-IHC (cell types) to determine cell-specific lipid spectra of the specific single cell types. On the subsequent MALDI-IHC measurements, three different conditions were prepared on the same ITO slide containing PDCL GBM single cells. This enables the investigation of the effect of prior MALDI-MSI sample preparation and measurements. One-third of the cells were stained with the Miralys probes without prior treatment (no prep), one-third went through the MALDI-MSI sample preparation treatment of matrix application but without the subsequent MSI measurement (MALDI prep) and the final third was prepared for and analyzed with MALDI-MSI (MSI1). An overview of the experimental setup can be seen in Supporting Figure S1. This setup would ensure that the compared sample preparations and cells were always the same between conditions. The results from the MALDI-IHC measurements can be seen in Figure 2.

Figure 2 shows data from the PDCL GBM single cells stained with MALDI-IHC. Using the optimized sample preparation workflow, single cells were able to be visualized and differentially stained with cell-specific antibody markers. Figure 2A shows the visualization of three PC-MTs, corresponding to antibodies targeting GLUT1 for GBM cells (*m*/*z* 856.67, red), NF-L for neurofilaments (*m*/*z* 1345.75, yellow) and SYN-I as a synaptic marker (*m*/*z* 1482.77, blue), with their respective single pixel mass spectra shown in 2B. Under normal conditions, GLUT1 is typically found in the blood-brain barrier as the transporter of glucose into the brain but has been found to be overexpressed in GBM cells likely due to the increased demand for glucose, and is therefore used as a marker for GBM cells here.^29−31^ An overview of all detected markers and corresponding average mass spectrum can be seen in Supporting Figures S3 and S4. Electron microscopy image and fluorescence image from immunostaining with GFAP can be seen in Supporting Figure S5. Each area of high intensity represents a single cell or cluster of cells. Note that each of the cell specific markers are present in different areas as expected by the cellular specificity of the antibodies in a heterogeneous single cell culture. Figure 2C–E shows images from test of pretreatment effects on the subsequent MALDI-IHC measurement. Figure 2C shows cells, untreated before MALDI-IHC, 2D shows cells that have undergone MALDI-MSI sample preparation, but no MALDI-MSI measurement, and 2E shows cells that have been prepared for and measured by MALDI-MSI. Imaging experiments were imported into the same data file, normalized to the root-mean-square, and visualized side-by-side with the same intensity for all data sets to compare any effects. No differences in intensity of MALDI-IHC probes were found between the three conditions. This was confirmed on a cell-by-cell basis by examining peak areas. For each condition, mean peak areas, per single cell, were compared between all measured MALDI-IHC targets, based on 10 cells per target and condition and showed no significant difference in intensity between conditions, Figure 2F. For the PC-MTs corresponding with NeuN, IBA-1, nicastrin and MAP2, the intensity was not sufficient to identify 10 single cells in the measured area in any of the three conditions. These results demonstrate how MALDI-MSI measurements prior to any MALDI-IHC imaging has no negative impact on the results, thus reinforcing how the two modalities can be easily combined to gain extra valuable information from a sample.

#### Multimodal Single Cell Correlation and Molecular Profiling

**Figure 3:**
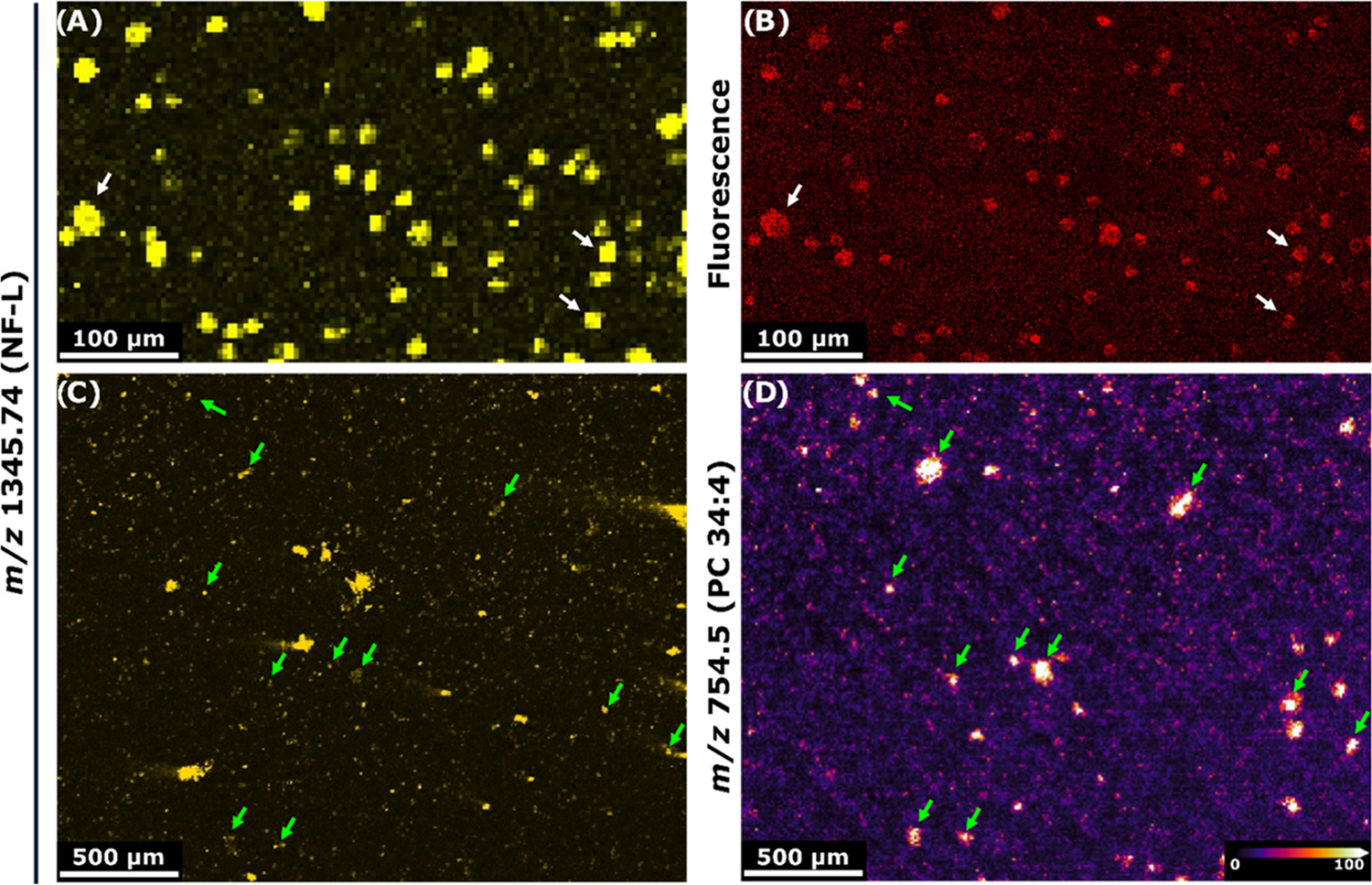
Multimodal imaging of single cells. (A–D) MALDI-IHC stained single cells (A + C), visualized with the marker for neurofilaments (NF-L) were correlated to images of the same cells obtained with fluorescence imaging (B) and MALDIMSI lipid imaging of *m*/*z* 754.5 (PC 34:4) (D). Two different field-of-views are visualized (A + B, C + D). Fluorescence images were detected in the range of 478–533 nm. MALDI-MSI images were obtained in positive-ion mode with a pixel size of 10 × 10 μm^2^. MALDI-IHC images were obtained at 5 × 5 μm^2^ pixel size. White and green arrows indicate correlating cells between images. Lipid IDs are based on LC-MS/MS from GBM tissue, as described in.^27^

The fluorescence capabilities of the Miralys probes were utilized to get high-resolution images of the stained cells and confirm that the high-intensity ion clusters were in fact single cells. This approach aids in the correlation of the different molecular imaging modalities, as demonstrated in Figure 3.

**Figure 4:**
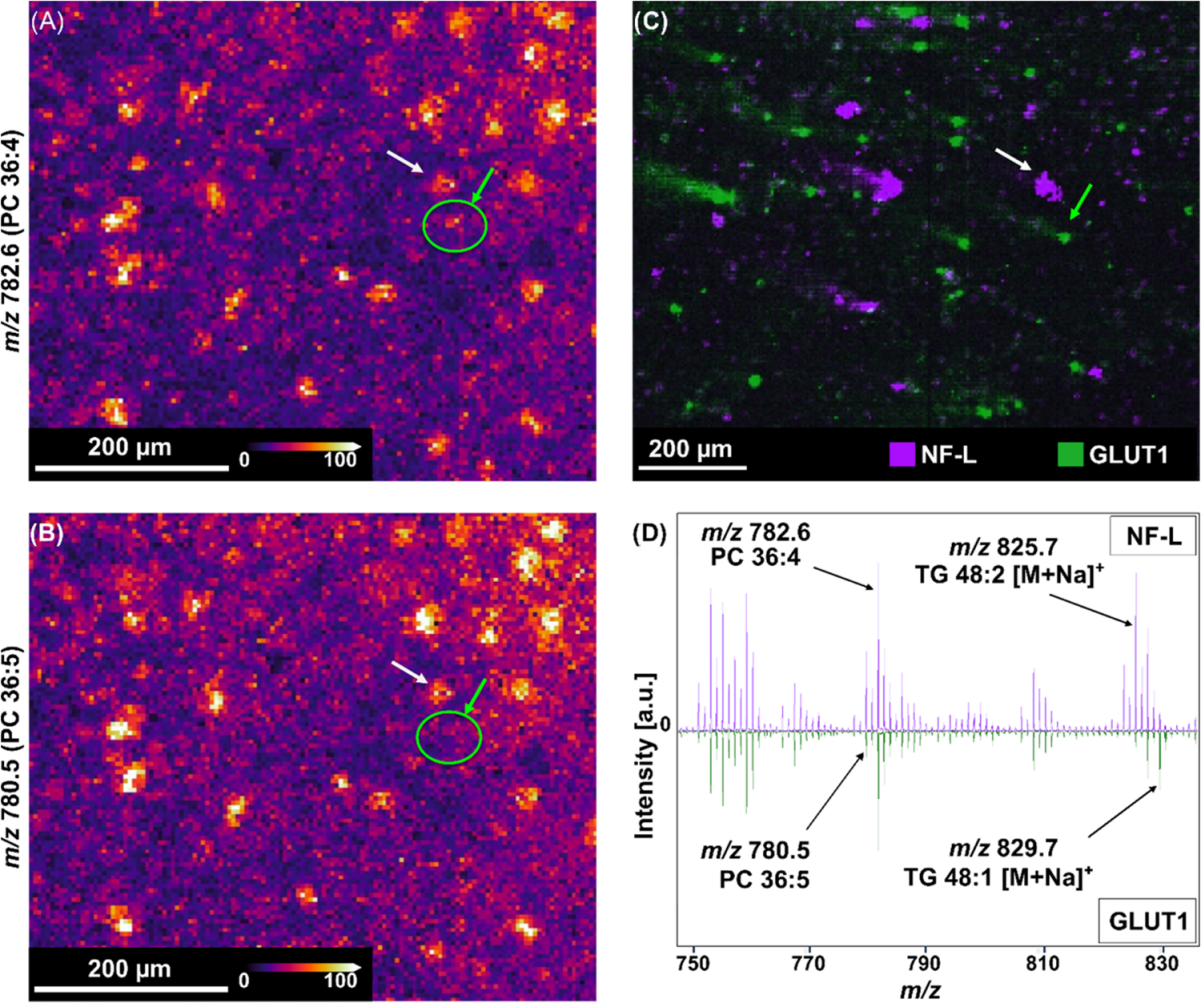
Molecular profiling of single cells. (A, B) MALDI-MSI images of lipid distributions in single cells. Pixel size = 10 × 10 μm. (C) MALDI-IHC image of single cells visualizing markers for GLUT1 (green) and NF-L (purple). Pixel size = 5 × 5 μm^2^. Single cells were correlated to lipid distributions in cells obtained with MALDI-MSI (white and green arrows). The white arrow indicates a cell correlating with the NF-L marker, while the green arrow indicates a cell correlating with GLUT1. The correlated cells show a different lipid expression profile, highlighted by *m*/*z* 780.5 (PC 36:5) not being detected in the GLUT1 expressing cell (green circle, (A, B)). (D) Mass spectra from each of the correlated cells with select masses highlighted as different between the spectra. Lipid IDs are based on LC-MS/MS from GBM tissue, as described in.^27^

Figure 3 showcases MALDI-IHC images corresponding with the PC-MT marker for neurofilaments (*m*/*z* 1345.74, NF-L) and therefore specifically the axons and dendrites in neurons (Figure 3A,C). Utilizing the fluorophore, also present on the Miralys probes, fluorescence images were also obtained of the same single cells as can be seen in Figure 3B. The cells were first imaged by MALDI-IHC and then by fluorescence imaging, allowing for precise overlay of the two modalities and it was therefore possible to find back many of the cells observed in MALDI-IHC, and in the fluorescence image as well, as indicated by the white arrows (Figure 3A,B). The fluorescence images obtained were not cell type-specific, as the fluorophore present on the Miralys probes used in the selected kit were the same for every probe. This meant that the fluorescence imaging could be used as a confirmation of a given antibody being bound to a single cell and that the signals observed in the corresponding MALDI-IHC images showed single-cell specificity. Furthermore, the MALDI-IHC images were correlated with the previously obtained MALDI-MSI lipid images of the same single cells (Figure 3C,D). The single cell-specific MALDI-IHC images were imported into the MALDI-MSI SCiLS datafile to precisely coregister the two measurements. This allowed for visualization of multiple lipids across specific cell types. Figure 3C shows an image correlating with the Miralys antibody for NF-L and Figure 3D visualizes the PC 34:4 at *m*/*z* 754.5, with some of the single cells that are recognizable in both images highlighted with green arrows. Comparing Figure 3C,3D, it is also clear that not all cells show up in both modalities with cells being uniquely present in both the MALDI-MSI and the MALDI-IHC images, respectively. When correlating the two modalities in two separate MALDI-IHC measurements, 54 of 396 (13.64%) and 17 of 144 (11.8%) of MALDI-MSI cells had no corresponding MALDI-IHC marker. A corresponding MALDI-IHC marker was included only when four or more pixels of high intensity were joined together. Furthermore, there are many cells where the intensity of lipids detected is not high enough to pick them out from the background, this is visualized in Supporting Figure S6. Finally, the apparent “streaking” of signal observed in larger clusters in the MALDI-IHC image (Figure 3C, right side) is likely due to insufficient drying following matrix sublimation. Supporting Figure S7 shows that cells are still intact postmeasurement, with only some MALDI-IHC markers presenting with streaks. Differences in lipid abundances between single cells of varying cell types, as classified by the MALDI-IHC data, were investigated, and are visualized in Figure 4.

Figure 4 shows how correlating MALDI-IHC and MALDI-MSI data can help with molecular profiling of single cells. Two MALDI-MSI lipid images from the same region are presented and show a discrepancy of the cell-distribution between different *m*/*z*-images. (Figure 4A,B). For the two cells/cell clusters highlighted, different lipid distributions are observed. A MALDI-IHC image of the same area shows the distribution of GLUT1 expressing cells (green) and neurofilaments in neurons (purple), with the same two single cells/cell clusters being highlighted by arrows (Figure 4C). While lipid PC 36:4 at *m*/*z* 782.6 is detected in both the GBM cell and the neuron, the other lipid, PC 36:5 at *m*/*z* 780.5, seems to only be present in the neuronal cell cluster and is not present in the GBM cell. This pattern can be seen in other areas in the MALDI-MSI images as well, with PC 36:5 not being present in all cells where PC 34:1 is present. The mass spectra from both selected cells are shown in Figure 4D, again showcasing the different lipid profile observed in both cell types and clearly highlights the intensity difference observed for PC 36:5, as well as differences observed in TG 48:2 and TG 48:1 between the two cells.

Additionally, it can be seen that fewer cell-signals are present in the MALDI-MSI images, compared to the MALDI-IHC images. This can be further accentuated when considering that only two out of 10 detected markers are visualized to reduce visual noise. This could indicate that the highly localized signals produced in MALDI-IHC, allows for higher sensitivity in smaller areas, compared to the complex lipid spectra that is present in each single cell and measured with MALDI-MSI. Furthermore, for these experiments, MALDI-MSI measurements were carried out with a pixel size of 10 × 10 μm^2^ whereas the MALDI-IHC measurements were done at 5 × 5 μm^2^. This could potentially result in loss of specificity for different lipids within the cells. Reproducing these experiments with both modalities at 5 × 5 μm^2^ would be interesting for a more direct comparison of images.

#### Cellular Recognition Modeling

**Figure 5:**
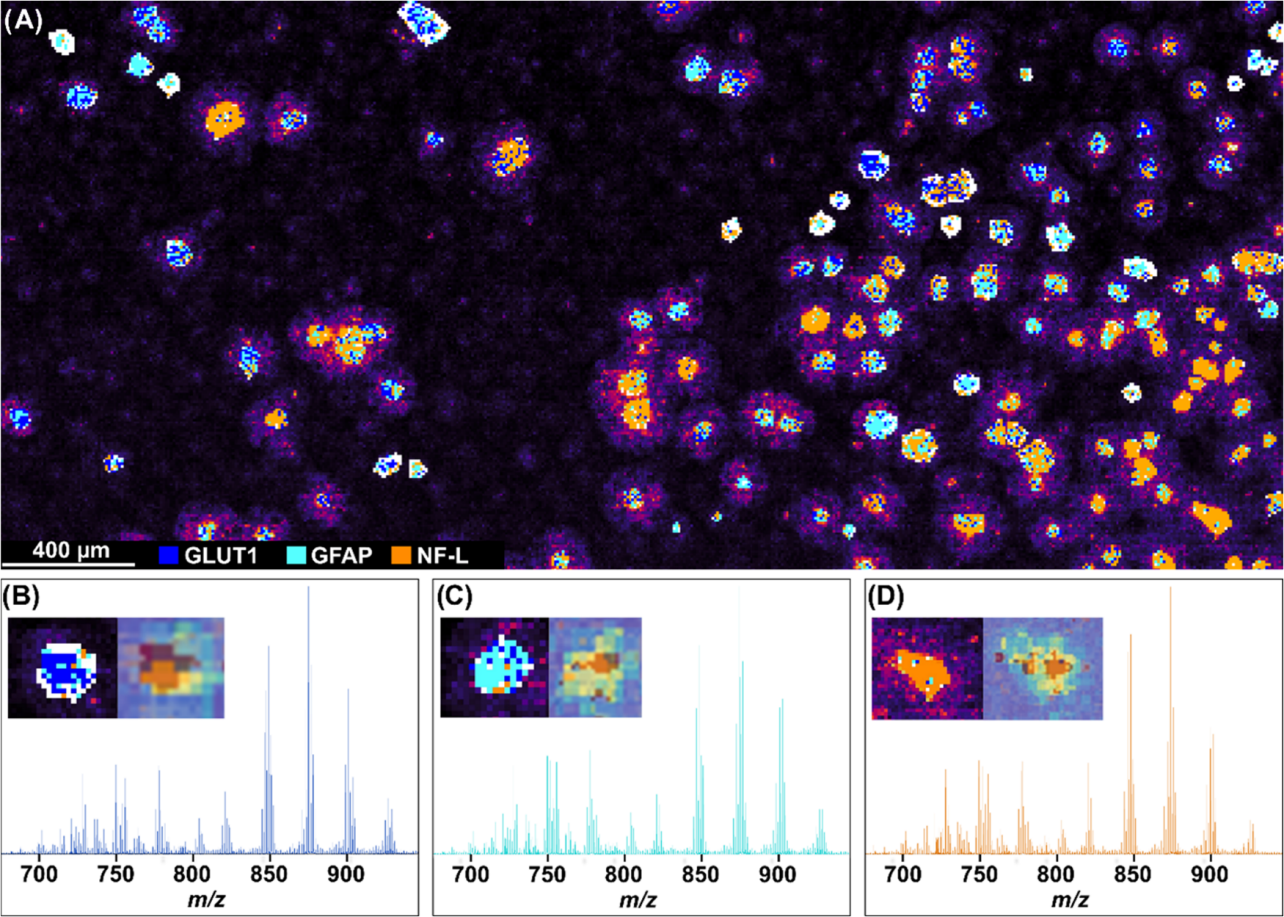
Automatic cellular recognition model of neuronal and glial cell types. (A) MALDI-MSI lipid distribution of single cells, with recognition model labels overlaid on each cell. Each cell-related ROI for the model was selected based on the lipid distribution. Blue pixels indicate a GLUT1-associated (GBM cells) spectrum. Cyan pixels indicate a GFAP-associated (astrocyte) spectrum. Orange pixels indicate an NF-L-associated spectrum. White pixels indicate poly-l-lysine-associated spectrum, indicative of background signal. (B–D) Cell type specific spectra from each recognized marker, respectively. For each cell type, an example of the recognition model overlay for a single cell is shown (left small image), as well as an overlay of the MALDI-IHC marker (cool-to-warm scale), on the MALDI-MSI (viridis-scale) image (right small image). Pixel size = 10 × 10 μm^2^.

A cellular recognition model was created based on the molecular profiles obtained by correlating the MALDI-MSI lipid data with the MALDI-IHC cell type analysis. Since each cell type showed a different lipid profile, these could be extracted on a per-cell basis to create a recognition model which automatically classified the cell type for a given MALDI-MSI spectrum. In this case, the model was built in SCiLS Lab on spectra associated with GLUT1 (GBM cells), GFAP (astrocytes), NF-L (neurons) and poly-l-lysine as a background. An overview of the recognition model overlaid on the corresponding MALDI-MSI measurement can be seen in Figure 5.

The overlay demonstrates that each pixel within a cell gets assigned to one of the four classes included in the model. For a cell to be classified to either one of the cell type classes, a threshold of 50% was chosen for class-associated pixels in a cellular ROI. The cell type assignment from the model was then compared to the cell type indicated by the MALDI-IHC staining. In total, 142 cellular ROIs were indicated in the MALDI-MSI lipid data set and used for the recognition model and 55 of these showed a clear correlation with a specific cellular marker in both the model and the corresponding MALDI-IHC image (Figure 5A). The rest of the ROIs did either not have a specific class with more than 50% pixels associated with it or did not correlate with any markers in the MALDI-IHC image. For each class, there was a false prediction rate of 22% (GLUT1), 17% (GFAP) and 6% (NF-L) on a per-cell basis, where a class was assigned to a cell based on the model, but the MALDI-IHC staining indicated a contrasting classification. Further investigation of the model showed that cells classified as neurons (>50% NF-L classified pixels), showed, on average, a higher percentage of NF-L specific pixels per cell (83%) versus GFAP assigned pixels for cells classified as astrocytes (57%) and GLUT1 assigned pixels for GBM cells (58%), based on five randomly selected cellular ROIs per class. Figure 5B–D shows the different lipid profiles observed between each cell type classification. The top ten contributing lipid *m*/*z* values for each class, based on the corresponding PCA, can be seen in Supporting Table S4. Notably, PC 36:4 is the third highest contributor to the GLUT1 class, while PC 36:5 is the third highest contributor to the NF-L class, in correspondence with the data shown in Figure 4.

This model works as a proof of concept for a cellular recognition model based on MSI lipid spectra alone, validated by MALDI-IHC cell-typing. Potentially, if a model was trained on enough robust data sets, the cell-typing obtained in this project could be done without the help of MALDI-IHC or alternative methods, thus reducing the overall work time. However, a number of points need to be considered beforehand. First, 62% of cells were classified as “mixed” by the model, with no specific class-associated pixel being more than 50% abundant. This could be due to the model-building software in SCiLS Lab, where there is no outlier option for pixels and all pixels are therefore forced into one of the included classes in the model. This will eventually lead to more false classifications. Another reason for this could be the overlap of expression between GBM cells and astrocytes. Some GBM cells originate from astrocytes, and the two cell types therefore share protein expression patterns and could be a potential explanation for the mixed cell classification. Moreover, the exact specificity and correlation with ion intensity of the probes used for MALDI-IHC is unknown. Knowing the total number of cells versus the number of cells to which the antibodies bound would give an indication of how effective the staining is. Additionally, when working with single cells, it is a point of discussion, when a positive signal correlates with a cell. On average the cells used in this project have a diameter of 30 μm, so when imaging with a spot size of 5 × 5 μm^2^, a positive signal could indicate that the antibody has bound to its target on a specific area of the cell, however that is also hard to conclude from one single pixel of high intensity. Finally, the cellular ROIs detected in the MALDI-MSI measurements are very large, when compared to the corresponding MALDI-IHC images and the expected size of the single cells. This can be explained by cells clustering on top of each other, thus appearing larger, or the difference in pixel-size between measurements. The larger pixel-size used in the MALDI-MSI measurement is more likely to pick up ions from multiple cells, and thereby indicate a larger area of ion intensity. Also, many cellular ROIs are present in the image at very low intensities, suggesting that the sensitivity of the method is not high enough to detect every single cell on the slide. However, the MALDI-MSI images also show a “lipid discharge” surrounding each cell, potentially masking smaller cells in close proximity of each other (Figure S8). In the future, conducting both the MALDI-MSI and the MALDI-IHC measurements with a pixel-size of 5 × 5 μm would potentially provide a better understanding of the cell-to-cell correlation between the two modalities.

## Conclusions

In conclusion, we developed an optimized workflow for multimodal imaging single cell samples using a 14-plex MALDI-IHC antibody panel—successfully detecting 10 out of 14 targets. Altering the matrix application technique and including a dip in ammonium phosphate monobasic, greatly increased sensitivity for detection of the MALDI-IHC probes at 5 × 5 μm^2^ spatial resolution. This allowed for cell type characterization and molecular profiling by correlating corresponding single cell MALDI-MSI measurements. Furthermore, we show that conducting MALDI-MSI on the single cell samples prior to MALDI-IHC staining and measurement, does not alter the observed intensity of the MALDI-IHC probes. Using this molecular profiling workflow, basic differences in lipid profiles between GBM cells and neurons were shown. The added information on altered lipidomic profiles obtained with MALDI-MSI could potentially help improve cell differentiation by adding the metabolic state to the suite of methods used for cell-typing. The ability of MALDI-MSI to distinguish single-cell-specific mass spectra across different cell types, further enabled the generation of a classification model which was successful in cell-typing of three different cell types using MALDI-MSI data alone. This proof-of-concept study shows how multiple imaging modalities can be used to extract single-cell data and eventually build large-scale recognition models for cell-typing by fully utilizing the strengths of MALDI-MSI.

## Supporting Information Available

Miralys probe information (Table S1); overview of cell treatment conditions prior to MALDI-IHC (Figure S1); replicate MALDI-IHC measurements taken during method optimization (Figure S2); overview of all detected PC-MTs with MALDI-IHC (Figure S3); average spectrum of MALDI-IHC measurement on unmeasured single cells (Figure S4); high-resolution images of unmeasured PDCL GBM single cells (Figure S5); visualization of low-intensity cells with MALDI-MSI (Figure S6); visualization of signal “streaking” observed in MALDI-IHC measurements (Figure S7); top 10 loadings per class used in the classification model (Table S4); MALDI-MSI images of cell-associated lipids and lipid “discharge” (Figure S8) (PDF)
Mean peak areas per cell (Table S2); mean peak areas per condition and statistics (Table S3) (XLSX)
