# Supplementary material for: Molecular Profiling of Glioblastoma Patient-Derived Single Cells Using Combined MALDI-MSI and MALDI-IHC
Source: Anal Chem. 2025 Feb 11;97(7):3846–54. doi: 10.1021/acs.analchem.4c03821 (PMC11866282; doi:10.1021/acs.analchem.4c03821)
Supplement: Supplementary file 1 — ac4c03821_si_001.pdf [file ac4c03821_si_001.pdf]

Supporting information:

Molecular profiling of glioblastoma patient-derived single cells using combined MALDI-MSI and MALDI-IHC

Kasper K. Krestensen[1], Tim F.E. Hendriks[1], Andrej Grgic[1], Marleen Derweduwe[2], Frederik De Smet[2], Ron M.A. Heeren[1], Eva Cuypers[1*]

[1]The Maastricht MultiModal Molecular Imaging (M4I) institute, Division of Imaging Mass Spectrometry (IMS), Maastricht University, 6229 ER Maastricht, The Netherlands
[2] Laboratory for Precision Cancer Medicine, Translational Cell and Tissue Unit, KU Leuven, 3001 Leuven, Belgium

ORCID
Kasper K. Krestensen https://orcid.org/0000-0003-0482-5205
Tim F.E. Hendriks https://orcid.org/0000-0003-2922-2953
Andrej Grgic https://orcid.org/0000-0002-4314-4456
Marleen Derweduwe https://orcid.org/0000-0002-4960-4847
Frederik De Smet https://orcid.org/0000-0002-6669-3335
Ron M.A. Heeren https://orcid.org/0000-0002-6533-717
Eva Cuypers https://orcid.org/0000-0002-6411-2461

* Corresponding author:
Eva Cuypers
Email: e.cuypers@maastrichtuniversity.nl

Table of Contents

Table S1: Miralys probe information 1

Figure S1: Overview of cell treatment conditions prior to MALDI-IHC 3

Figure S2: Replicate MALDI-IHC measurements taken during method optimization 4

Table S2: Mean peak areas per cell. *See SI excel*. 5

Table S3: Mean peak areas per condition and statistics. *See SI excel*. 5

Figure S3: Overview of all detected PC-MTs with MALDI-IHC..........................6

Figure S4: Average spectrum of MALDI-IHC measurement on unmeasured single cells. .7

Figure S5: High-resolution images of unmeasured PDCL GBM single cells 8

Figure S6: Visualization of low-intensity cells in MALDI-MSI 9

Figure S7: Visualization of signal 'streaking' observed in MALDI-IHC measurements....10

Table S4: Top 10 loadings per class used in the classification model...............11

Figure S8: MALDI-MSI images of cell-associated lipids and lipid 'discharge'...........12

Table S1: Miralys probe information used for MALDI-IHC experiments

Binding agent	Concentration	Working volume (2 µg/mL)	PC-MT (*m/z*)	Species reactivity*	Detected
**GLUT1**	250 µg/mL	9.6 µL	856.56	M, R, H	Yes
**GFAP**	250 µg/mL	9.6 µL	1011.55	M, R, H	Yes
**SNCA**	250 µg/mL	9.6 µL	1045.57	M, R, H	Yes
**pTau-(pS404)**	250 µg/mL	9.6 µL	1201.69	M, R, H	Yes
**NeuN**	250 µg/mL	9.6 µL	1308.71	M, R, H	No
**NF-L**	250 µg/mL	9.6 µL	1345.74	M, R, H	Yes
**MBP**	250 µg/mL	9.6 µL	1365.73	M, R, H	Yes
**SYN-I**	250 µg/mL	9.6 µL	1482.77	M, R, H	Yes
**PVALB**	250 µg/mL	9.6 µL	1539.79	M, R, H	Yes
**CD163**	386 µg/mL	6.22 µL	1546.73	H	Yes
**IBA-1**	354 µg/mL	6.78 µL	1551.85	M, R, H	No
**MAP2**	349 µg/mL	6.9 µL	1569.80	M, R, H	No
**Nicastrin**	388 µg/mL	6.2 µL	1573.75	M, R, H	No
**Amyloid-β42**	250 µg/mL	9.6 µL	1770.88	H	Yes

*M = Mouse, R = Rat, H = Human

Main cell type presence

GLUT1 – Glioblastoma cell
GFAP – Astrocyte
SNCA – Neuron
pTau-(pS404) – Neuron, glia cells, astrocyte
NeuN – Postmitotic neuron
NF-L – Neuron
MBP – Oligodendrocyte
SYN-I – Synaptic marker, neuron
PVALB – GABAergic interneuron, Purkinje cell
CD163 – Activated macrophage
IBA-1 – Activated microglia
MAP2 - Neuron
Nicastrin – Neuron
Amyloid-β42 - Neuron

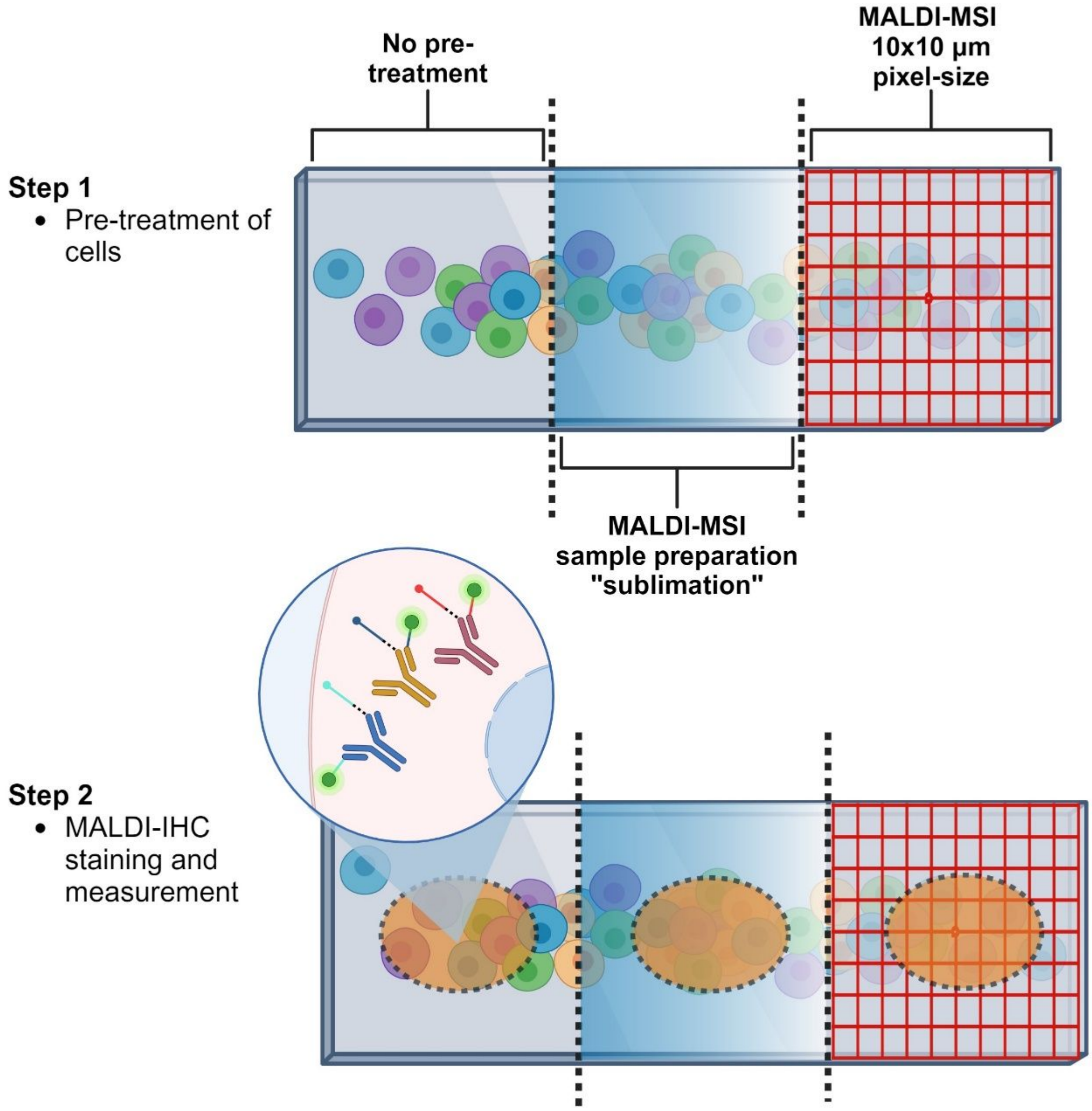

Figure S1: Overview of cell treatment conditions prior to MALDI-IHC. Cells either had no pre-treatment, underwent MALDI-MSI sample preparation with no MALDI-MSI measurement, or MALDI-MSI sample preparation followed by measurement. Following each condition, cells were stained and measured with MALDI-IHC. Created in BioRender.com.

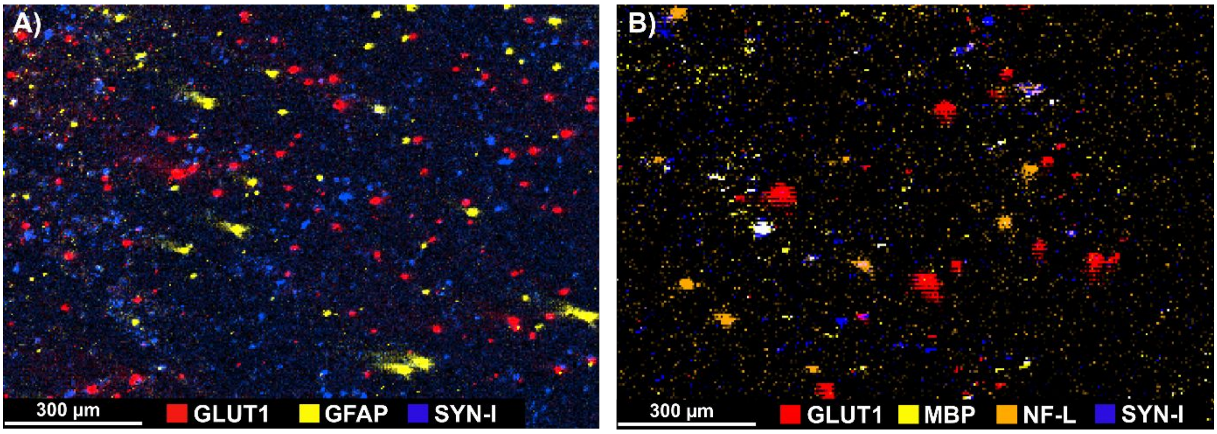

Figure S2: Replicate MALDI-IHC measurements taken during method optimization. (A) Three different cell markers are highlighted: GLUT1, for GBM cells, in red, GFAP for astrocytes, in yellow and SYN-I, as a synaptic marker, in blue. (B) Four different cell markers are highlighted: GLUT1, for GBM cells, in red, MBP for myelin in oligodendrocytes, in yellow, NF-L for neurofilaments in neurons, in orange and SYN-I, as a synaptic marker, in blue. Pixel size = 5x5 µm. (A) and (B) represent two different ITO slides measured.

Table S2: Mean peak areas per cell. *See SI excel*.

Table S3: Mean peak areas per condition and statistics. *See SI excel*.

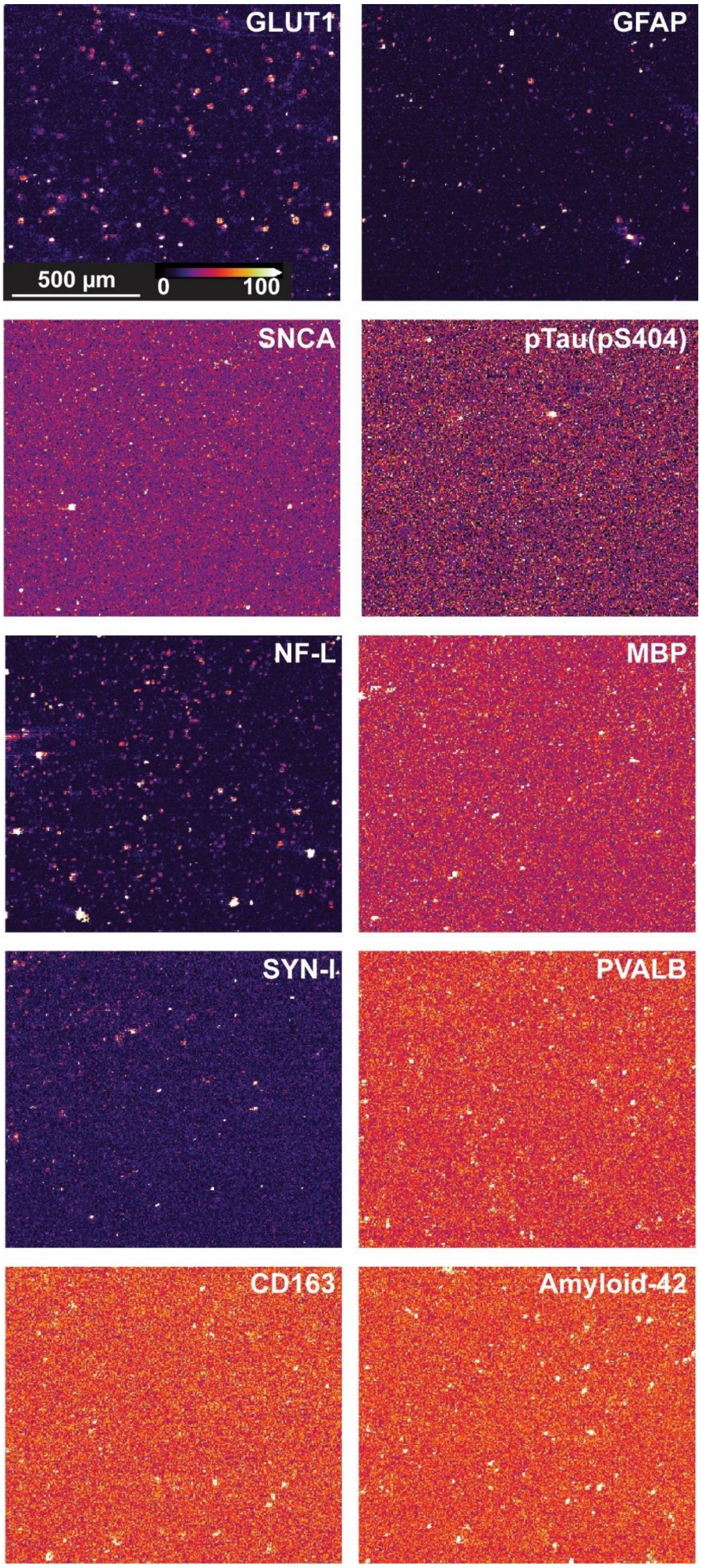

Figure S3: Overview of all detected PC-MTs with MALDI-IHC. 10 of 14 total markers were detected with significant intensity to pick out cell-like regions from the background signal. Pixel size = 5x5 µm.

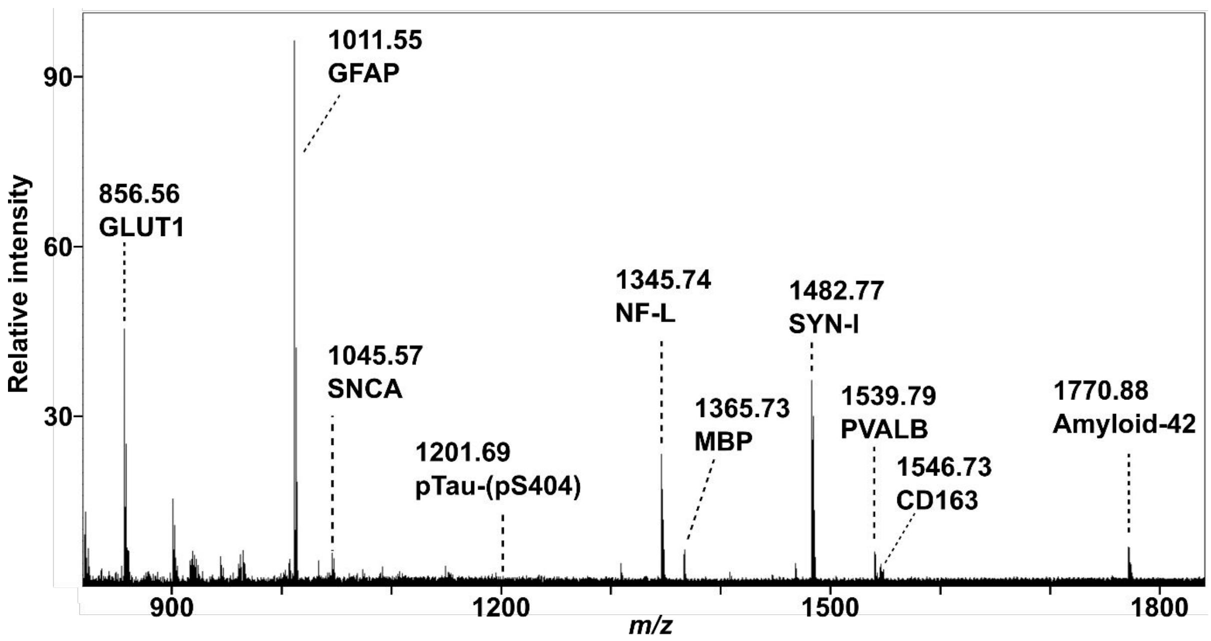

Figure S4: Average spectrum of MALDI-IHC measurement on unmeasured single cells. Corresponding MALDI-IHC images can be seen in figure S2 for each of the detected markers. In total, 10 out of 14 markers (labelled in figure) were detected with significant intensity to pick out cell-like regions from the background signal.

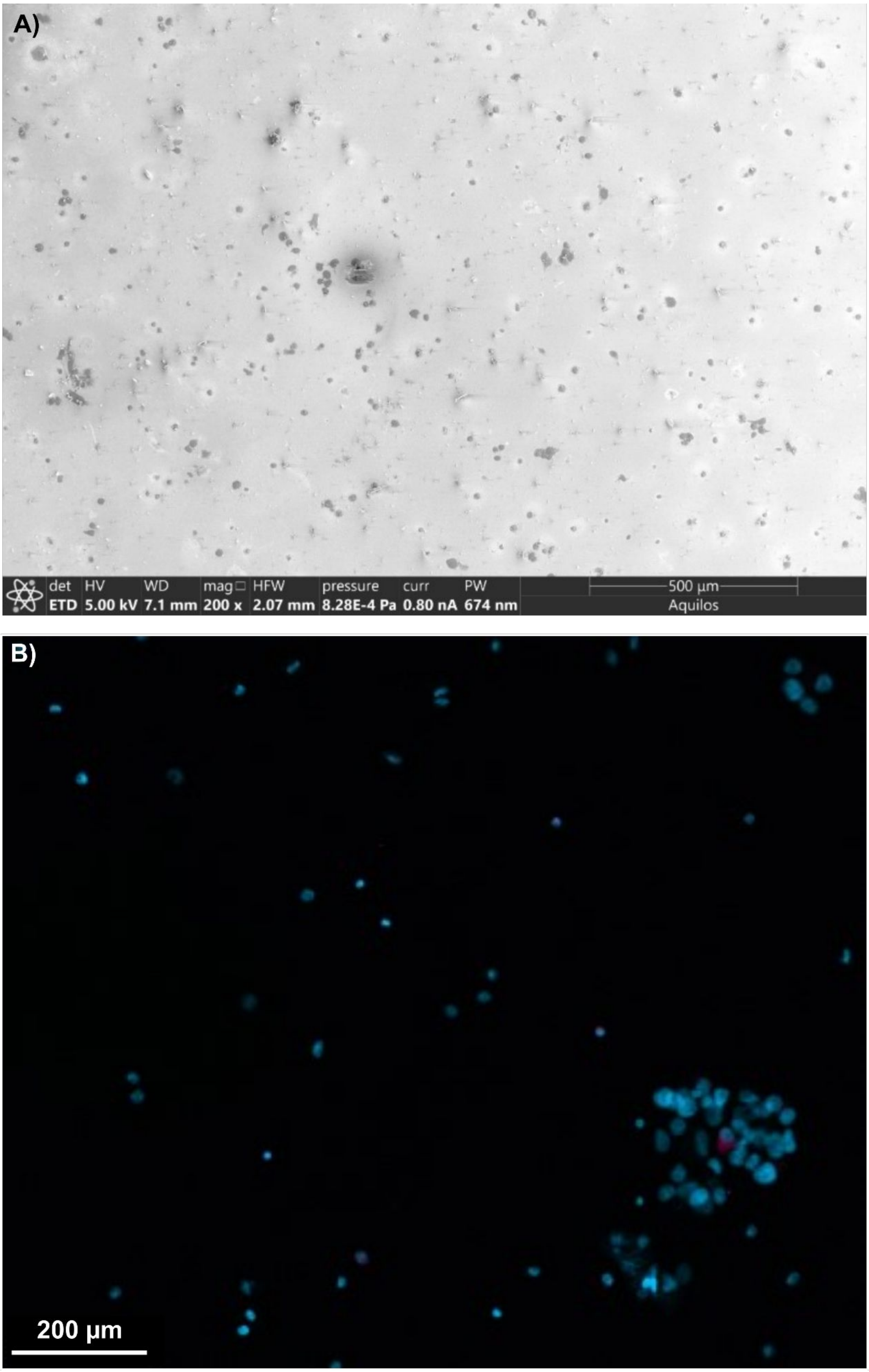

Figure S5: High-resolution images of unmeasured PDCL GBM single cells. (A) Electron microscope image of single cells on a glass slide. (B) Single cells stained with a conventional GFAP antibody and imaged.

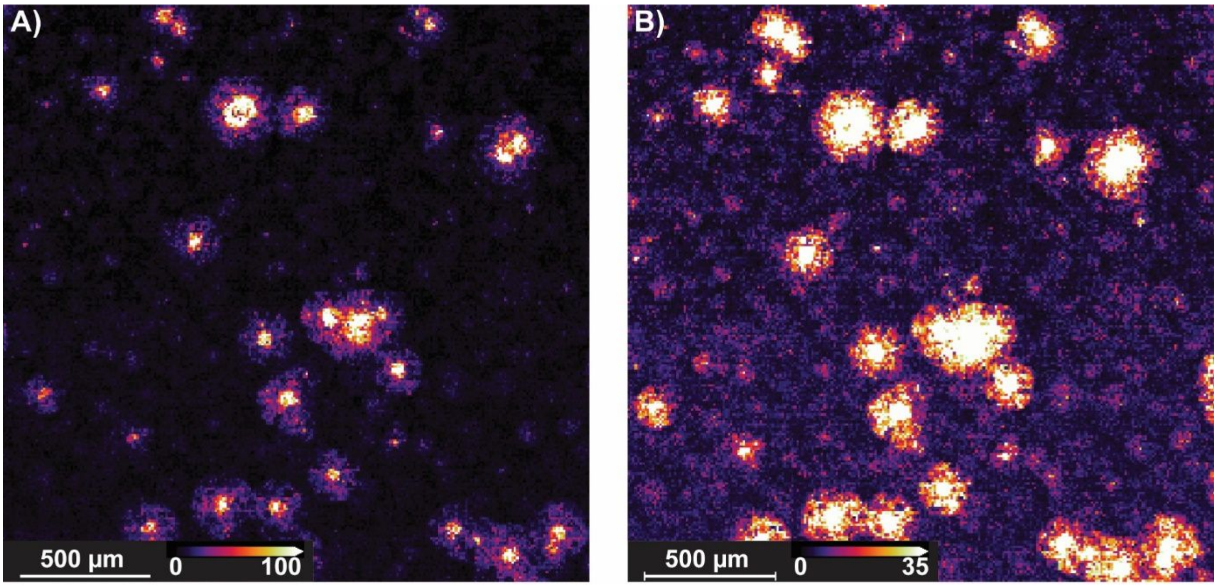

Figure S6: Visualization of low-intensity cells in MALDI-MSI. (**A**) MALDI-MSI lipid distribution of *m/z* 881.75 (TG 52:2) with intensity scale set to 100 %. (**B**) MALDI-MSI lipid distribution of *m/z* 881.75 (TG 52:2) with intensity scale set to 35 %, visualizing abundantly more cells in the same area. Pixel size = 10x10 µm.

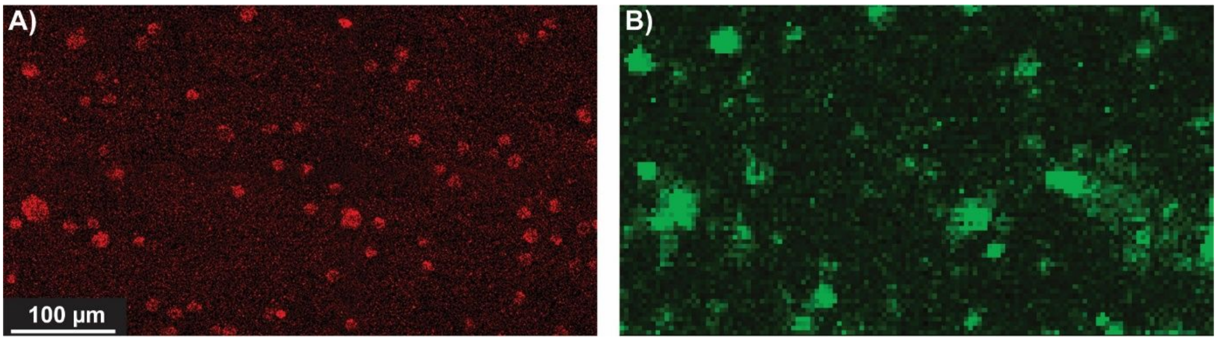

Figure S7: Visualization of signal 'streaking' observed in MALDI-IHC measurements. (**A**) Fluorescence image of stained and imaged PDCL GBM cells. (**B**) MALDI-IHC image of single cells visualizing marker for GLUT1. Streaking surrounding some cells is evident. Pixel size = 5x5 µm.

Table S4: Top 10 loadings per class used in the classification model are listed, with the corresponding lipid ID. Lipid IDs are based on LC-MS/MS from glioblastoma tissue.

**GLUT1**	
**Loading**	**ID**
5.653	TG(56:7) [M+H]+
5.340	TG 52:2 [M+Na]+
3.299	PC(36:4) [M+H]+
2.824	PC(34:4) [M+H]+
2.342	TG(48:1) [M+NH4]+
2.206	SM(d18:2/20:1) [M+H]+
2.204	N/A
2.204	N/A
2.179	TG(55:1) [M+Na]+
2.078	TG(51:5) [M+Na]+
**GFAP**	
**Loading**	**ID**
5.439	TG 52:2 [M+Na]+
3.039	SM(d18:2/20:1) [M+H]+
2.731	TG(56:9) [M+H]+
2.380	SM(41:2) [M+H]+
2.358	N/A
1.963	TG(52:4) [M+Na]+
1.795	TG(55:4) [M+H]+
1.755	N/A
1.748	DG(16:1/20:3/0:0) [M+H]+
1.739	PE 38:5 [M+H]+
**NF-L**	
**Loading**	**ID**
5.419	TG(52:5) [M+H]+
4.989	TG(50:3) [M+Na]+
2.528	PC(36:5) [M+H]+
2.387	PC(32:0) [M+H]+
2.055	DG(18:1/20:4/0:0) [M+H]+
1.991	N/A
1.960	SM(34:0) [M+H]+
1.917	PE(P-40:6) [M+Na]+
1.885	TG(60:10) [M+H]+
1.870	SM(38:0) [M+H]+

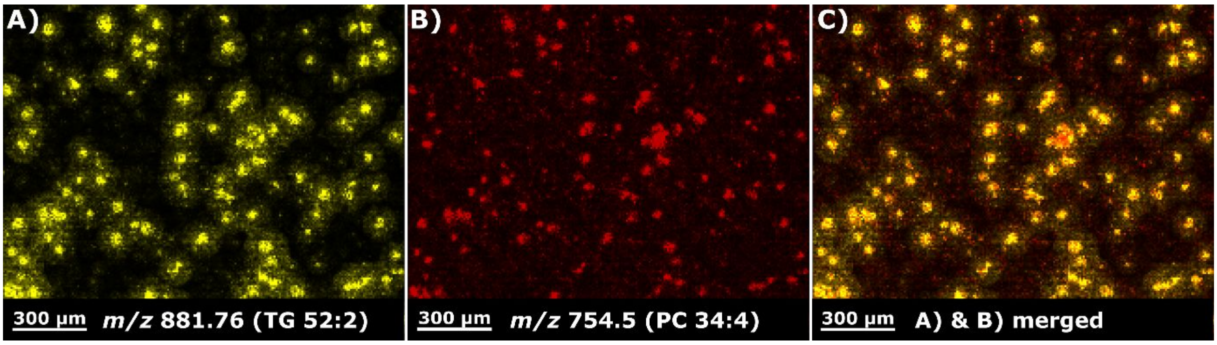

Figure S8: MALDI-MSI images of cell-associated lipids and lipid ‘discharge’. MALDI-MSI lipid distributions of *m/z* 881.75 (TG 52:2) (**A**), *m/z* 754.5 (PC 34:4) (**B**) and a merge of the two ion images (**C**). Focusing on the single cellular signals, a clear ring of lower intensity is present around each cellular ROI correlating with TG 52:2 (**A**), while PC 34:4 (**B**) shows a more contained spatial localization. Pixel size = 10x10 µm.
